# Further Critical Reflections on the Notion of a “Population Psychology”

**DOI:** 10.17505/jpor.2024.27103

**Published:** 2024-12-13

**Authors:** James T. Lamiell

**Affiliations:** Psychology Department, Georgetown University, Washington D.C., USA

**Keywords:** population psychology, psycho-demography, person psychology, mechanism psychology, causation

## Abstract

The present article extends critical considerations that I offered in an earlier article challenging the proposition by Lundh that population-level research should be regarded as a branch of psychological science. It is again acknowledged that population-level research can track the incidence of psychological phenomena, i.e., sensations, perceptions, judgments, cognitions, emotions, behaviors, etc., within and across various subgroups of individuals, and this, I argue, is what warrants the designation of such research as “psycho-demographic.” Such research can complement, but should not be considered part of, psychological science. It is explained that this view does not require strict adherence to a mechanistic understanding of causation in the domain of psychological phenomena. Finally, it is suggested that drawing and maintaining a clear distinction between psycho-demography and psychological science will help to correct the long-prevalent but false notion that the knowledge produced by population-level research is interpretable as knowledge about individuals.

In a highly thoughtful article published in 2023, Lars-Gunnar Lundh proposed that contemporary scientific psychology be formally recognized as having three main branches, where the research focus would be, respectively, on persons, on populations, and on the mechanisms underlying psychological phenomena (Lundh, 2023). At the request of Lundh, I authored a brief critical commentary on his proposal in which I expressed agreement with his identification of distinct research domains concerning (a) the psychological doings of persons as such, on the one hand, and (b) the neurophysiological bases of those doings, on the other hand (Lamiell, 2024a). I questioned, however, the conceptual integrity of the notion of “population psychology.” The central thesis of my argument in this regard is that while the ultimate objective of a genuinely *psychological* science is knowledge of individual-level realities, population-level studies do not and cannot provide such knowledge. Unconvinced, Lundh (2024) has authored another article in which he has rejoined my argument. In this article, I address myself to his rejoinder.

## Further on the Notion of a “Population Psychology”

In terms of its essential features, what Lundh wishes to call “population psychology” is what I have chosen to call “psycho-demography” (cf. Lamiell, 2019). Lundh, however, has challenged my characterization of such work as a species of demography on the grounds that demographic inquiry is “commonly defined as the statistical study of populations in terms of their size, composition (e.g., age, ethnicity) and how they change due to fertility, mortality, and migration” (Lundh, 2024, p. 71, parentheses in original). However, in accordance with the second edition of *Webster's New World Dictionary*, published in 2015, I understand demography to be *the study of populations*, full stop. The question “in terms of what?” is left open. However “common” it may be for demographic studies to concern such phenomena as those mentioned by Lundh (2024), it remains the case that what makes demographic studies *demographic* is that they are studies of *populations*.

Putatively “psychological” research designed to investigate statistical relationships between variables defined only for aggregates of individuals—i.e., populations, whether real or hypothetical—is research of an essentially demographic nature. That neither psychologists nor other social scientists nor the lay public “commonly” refer to such studies as “demographic” changes nothing. Their epistemic nature is defined by their methods and by the kind of knowledge that those methods produce. Their methods inherently entail the aggregation of information across individuals, and the resulting knowledge is knowledge of the aggregates *as such*.

Contrary to Lundh’s conjecture, I *do* claim that these considerations apply to “sociological” studies and, yes, also to research in the discipline commonly referred to as “population genetics.” Strictly speaking, the knowledge provided by “population genetics” is *not* knowledge of genetics, which is a sub-specialty of biology, but is, rather, knowledge defined by some statistical feature of a genetically determined phenomenon within a population. To provide a simple example: knowledge about the relative frequency of blue eyes in Norway as opposed to Italy is not knowledge about the genetics of eye color. It is knowledge about the frequency with which designated eye colors occur within different populations. On this view, the disciplinary label “population genetics” is as much of a misnomer as is “population psychology.”

In my 2019 book, *Psychology's Misuse of Statistics and Persistent Dismissal of its Critics*, I elaborate on my invocation of the term “psycho-demography” as follows:

Psycho-demography is a discipline that is *nominally* psychological in that the variables defined for investigation reflect a theoretical interest on the part of investigators in the psychological doings of individuals. Nevertheless, the discipline is *essentially* demographic because its paradigmatic statistical methods are suited only to the production of knowledge about populations. (Lamiell, 2019, p. 18)

Lundh (2024) states clearly his agreement with me on the point that population-level research and individual-level research “provide very different kinds of knowledge” (p. 71). He goes on to argue, however, that since “both kinds of knowledge are important to the development of psychological science” (p. 71, emphasis added), a statement with which I can agree, it is fair to say that “knowledge of population-level associations between psychological phenomena *also* represents an important form of psychological knowledge” (p. 71, emphasis in original). This latter claim is one with which I cannot agree.

Putatively “psychological” studies of the sort we are discussing are “psychological” only in the sense that the variables chosen for population-level investigation refer to phenomena widely regarded as psychological in nature (sensations, perceptions, judgments, cognitions, memories, emotions, behaviors, etc.). Although the results of population-level studies might very well *point in the direction* of truly psychological investigations—meaning, minimally, that those investigations would yield individual-level knowledge of psychological phenomena—the population-level studies themselves yield knowledge of *no one*, and I simply cannot accept the proposition that knowledge of no one qualifies as *psychological* knowledge—causal or otherwise (more on causation below).

As stated above, the name I have given to such studies, “psycho-demography,” acknowledges that the theoretical interests of investigators are often in knowledge of a psychological sort. But in juxtaposing the hyphenated terms of the expression “psycho-demography” as I have, I have quite deliberately stamped those studies themselves as demographic in nature, because that is exactly what they are. What they are *not* are studies that are *themselves* generative of *psychological* knowledge, and that is why I regard the label “population psychology” as unwarranted and misleading.

The formal suitability of population-level studies for generating statistical knowledge of individuals’ “risk factors for,” or of their “degree of predictability” with respect to various events or conditions, or their susceptibility to causal influence by certain treatments, is to many what qualifies such studies as “psychological” (for a recent example of this, see Proctor and Xiong, 2018; cf. Lamiell, 2018a, 2018b). Lundh (2024) seems to implicitly accept this view in, for example, his discussion, beginning on p. 69, of causality in psychotherapy research.

In any case, this view is epistemically unsound, because the notion that population-level studies are suited to generating knowledge of the sort just indicated, whatever degree of uncertainty or probability it might declare, is erroneous. In Chapter 5 of my aforementioned 2019 book (Lamiell, 2019), I explain at length why this is so, and the interested reader is referred to that source. In consideration of space constraints here, the discussion’s bottom line must suffice: population-level statistical knowledge of “extent of risk” or “degree of predictability” or “susceptibility to causal influence”—is *not* knowledge of any particular person’s risk or degree of predictability or susceptibility to some causal influence on his/her behavior. Population-level knowledge simply cannot validly be made into knowledge about individuals, and adopting probabilistic language to express that knowledge cannot alter this logical fact. That is just the way it is.

## Further on the Matter of Causality

In my previous critical commentary on the notion of a “population psychology” (Lamiell, 2024a), I questioned Lundh's (2023) claim that causal structures involving psychological phenomena could be operative at the population level. In doing so I appealed to the observation by the philosopher Rom Harré (1927-2019) that “mechanisms of action must be realized in particular persons, … even when they are acting as members of collectives” (Harré, 1981, p. 14). Lundh (2024) has rejoined that argument by rejecting what he views as an exclusive commitment on my part to a strictly mechanistic understanding of causation.

However, my objection to the notion of a “population psychology” does not logically require an appeal to a *purely* mechanistic conception of causation. On the contrary, it is quite possible for my argument to incorporate a teleological component as well into psychological explanations for individual doings, and so to include Aristotelian “final” cause statements in the form of references to a person’s intentions (Rychlak, 1981). What my argument does require, however causation might be conceived, is that causal explanations in any discipline worthy of the name “psychology” be intelligible *at the level of the individual* (this for reasons mentioned above).

For more than 30 years now, I have been studying the works of the German philosopher and psychologist William Stern (1871-1938), who was firmly committed to an agentic conception of individual persons, and hence to incorporating reference to persons’ *intentions* into the formulation of scientific explanations for their doings (cf. Lamiell, 2021, 2024b). Stern’s “critically personalistic” perspective is one with which I fully agree. That said, it must also be noted that the incorporation of intentionality into causal explanations for psychological phenomena neither obviates reference to “material” and “efficient” causes, nor does it necessitate or somehow legitimize the notion of “population-level causation” for psychological phenomena (cf. Rychlak, 1988).

In my view, it is metaphysically problematic to suppose that intentions can somehow exist as forces operative in a kind of conceptual ether, somehow causally consequential yet existing for no one in particular. On the contrary, where there are causes for phenomena of theoretical interest to psychologists—possibly including a person’s intentions, conscious or otherwise—there must be *effects*, and some theoretical account for the occurrence of those causes and realization of their effects at the level of individual persons must be provided. Within the conceptual framework Stern called “critical personalism,” he developed just such a theoretical account. He wrote of the *convergence* (*die Konvergenz*) of the person with his/her social world by means of the critical adoption or “introception” (*die Introzeption*) into his/her own value system of certain of the values held by others (cf. Lamiell, 2021, 2024b). Stern fully appreciated that for the purposes of a genuinely psychological science, causes and effects must transpire *at the level of individual persons*, and, as Harré (1981) also clearly saw, this is true no matter how widely those same causes and effects transpire across the individuals located within some given population/culture/historical epoch.

As Lundh (2024) has properly noted, population-level investigations such as randomized controlled trials (RCTs) and studies of non-suicidal self-injury (NSSI) can suggest possibly fruitful directions for studies that are formally suited to uncovering such cause-effect relationships at the level of the individual. In Lundh’s view, it would appear (cf. Lundh, 2024, p. 70), this suffices to regard population-level investigations as *part of* psychological science, whereas in my view such investigations are better regarded as *possible complements to* psychological science. While I can readily concede that the line of distinction here is a fine one, I find it worth maintaining because it underscores the point, one that has been systematically ignored over many decades by several generations of researchers (cf. Lamiell, 2019), that the population-level statistical findings generated by the research methods—experimental, correlational, and/or hybrid—that have long since become standard within the mainstream are *not* validly interpretable *at all* as knowledge about individual-level doings. I believe that, going forward, the label “psycho-demography” will highlight this epistemically crucial point better than will the label “population psychology.”

In Chapter 7 of Lamiell (2019), the reader will find substantial space devoted to discussing the potential usefulness of psycho-demographic inquiry, both in its own right as a practical guide to public policy, and as a prompt to further inquiry of a genuinely psychological nature. Also discussed in that work is one methodological framework within which such inquiry can proceed. The framework is called by its developer, James W. Grice (born 1964), “observation-oriented modeling” (see, e.g., Grice, 2011, 2015). The vision of psychological science advanced in these relatively recent works can readily incorporate the distinction Lundh (2023, 2024) has drawn between “person psychology” and “mechanism psychology,” and would be nicely complemented by the kind of psycho-demographic work that Lundh has called “population psychology.” It is to be hoped that these possibilities, appropriately named, will be widely recognized and pursued as such in the coming years.

## Action editor

Lars-Gunnar Lundh served as action editor for this article.

## Open access

This article is distributed under the terms of the Creative Commons Attribution 4.0 International License (http://creativecommons.org/licenses/by/4.0/), which permits unrestricted use, distribution, and reproduction in any medium, provided you give appropriate credit to the original author(s) and the source, provide a link to the Creative Commons license, and indicate if changes were made.
